# The Importance of Animal Models in Biomedical Research: Current Insights and Applications

**DOI:** 10.3390/ani13071223

**Published:** 2023-03-31

**Authors:** Adriana Domínguez-Oliva, Ismael Hernández-Ávalos, Julio Martínez-Burnes, Adriana Olmos-Hernández, Antonio Verduzco-Mendoza, Daniel Mota-Rojas

**Affiliations:** 1 Master’s Program in Agricultural and Livestock Sciences [Maestría en Ciencias Agropecuarias], Xochimilco Campus, Universidad Autónoma Metropolitana (UAM), Mexico City 04960, Mexico; 2 Clinical Pharmacology and Veterinary Anesthesia, Facultad de Estudios Superiores Cuautitlán, Universidad Nacional Autónoma de México (UNAM), Cuautitlán 54714, Mexico; 3 Facultad de Medicina Veterinaria y Zootecnia, Universidad Autónoma de Tamaulipas, Victoria City 87000, Mexico; 4 Division of Biotechnology—Bioterio and Experimental Surgery, Instituto Nacional de Rehabilitación-Luis, Guillermo Ibarra Ibarra (INR-LGII), Mexico City 14389, Mexico; 5 Neurophysiology, Behavior and Animal Welfare Assessment, DPAA, Universidad Autónoma Metropolitana (UAM), Mexico City 04960, Mexico

**Keywords:** translational research, animal research, laboratory animals, rodents, primates, pigs, zebrafish, nematodes

## Abstract

**Simple Summary:**

The present review highlights and examines the importance of animal models in relevant topics concerning current human and animal health. Over the past five years, different animal species have been used to study pandemics, such as the 2019 Coronavirus, diabetes, and obesity. Through murine, primate, porcine, and even aquatic models (e.g., zebrafish), several neurological, behavioral, cardiovascular, and oncological disorders are being understood while developing new therapeutic approaches. Nematodes and arthropods are some of the new alternatives for biomedical science; however, regardless of the species, many animal research studies show the vital role of animal models in advancing biomedical research.

**Abstract:**

Animal research is considered a key element in advance of biomedical science. Although its use is controversial and raises ethical challenges, the contribution of animal models in medicine is essential for understanding the physiopathology and novel treatment alternatives for several animal and human diseases. Current pandemics’ pathology, such as the 2019 Coronavirus disease, has been studied in primate, rodent, and porcine models to recognize infection routes and develop therapeutic protocols. Worldwide issues such as diabetes, obesity, neurological disorders, pain, rehabilitation medicine, and surgical techniques require studying the process in different animal species before testing them on humans. Due to their relevance, this article aims to discuss the importance of animal models in diverse lines of biomedical research by analyzing the contributions of the various species utilized in science over the past five years about key topics concerning human and animal health.

## 1. Introduction

The use of animals in scientific research is controversial [1]. However, the transformation of medicine from an art to a science can be mainly attributed to using a wide range of animal models [2], selected according to their functional and genetic characteristics for specific research lines [3]. Animal models contribute significantly to the advance of biomedical science through their meaningful contributions to our growing understanding of pathological and biological processes [4]. Moreover, they enable the development and testing of drugs, vaccines, and surgical techniques applicable to human and veterinary medicine [5].

The term “animal model” comes from the Latin *animae* (alma or spirit) and the word model, which means to imitate or be similar to [6]. Animal models are based on the principle of comparative medicine [7] as instruments that can replicate physiological and pathological processes [8]. The species is selected according to each project’s objective and hypothesis [3] but also considers biological, anatomical, functional, and genetic similarities to humans or other animals [6]. Today, most of the species utilized in biomedical research are rodents [9], as they are deemed ideal models for studying pathologies that affect human populations due to their physiological homology [10], which allows them to be employed to further our understanding of such processes as sepsis, obesity, cancer, organ transplants, and biological development, among many others [11,12].

The species used in experimentation are not limited to small mammals. Rhesus monkeys (*Macaca mulata*) are utilized to study high-priority diseases such as the pandemic caused by the severe, acute respiratory syndrome type 2 coronavirus (SARS-CoV-2) [13]. Domestic pigs (*Sus scrofa*) are crucial for organ transplant medicine and immune therapies [14]. New species, including some invertebrates such as fruit flies (*Drosophila melanogaster*), are used to study neurological disorders such as epilepsy [15], nematodes such as *Caenorhabditis elegans* to study obesity [16], and aquatic models, such as the zebrafish (*Danio rerio*), to treat metabolic disorders, including diabetes [17].

The broad range of species used in research has brought exponential advances in medicine, especially with the introduction of genetically modified (transgenic) animals [18] and the implementation of supporting technologies such as nanotechnology and artificial intelligence [19]. In light of this, this article aims to discuss the importance of animal models in diverse lines of biomedical research by analyzing the contributions of the various species utilized in science over the past five years concerning key topics of human and animal health.

## 2. Search Methodology

The literature search was performed in the Web of Science, Scopus, and PubMed. Keywords related to the use of animal models applied to current research priorities were searched to select the relevant articles, for example, “emerging infectious disease”, “diabetes and obesity”, “neurodegenerative diseases”, “pain therapies”, “surgical techniques”, “cancer models”, and “alternative animal models”. The search was limited to articles published in English in the last five years (2019–2023) and related to human and non-human medicine and therapeutics.

## 3. A Review of Animal Experimentation

**Figure 1:**
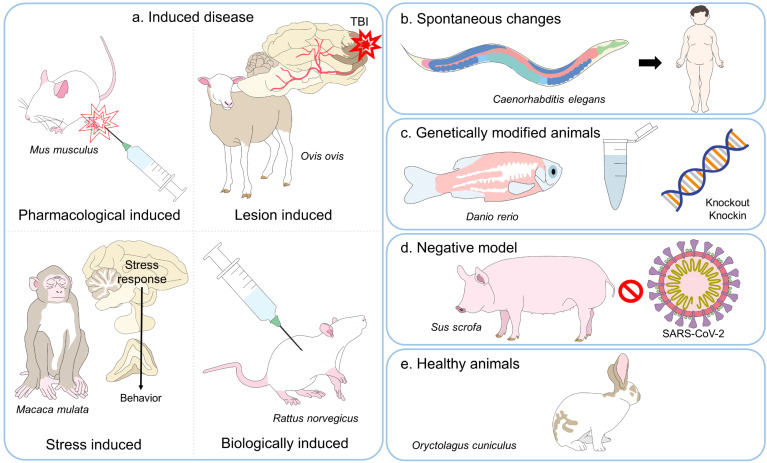
Classification of various animal models. The animals used in science can be divided into five broad types. (**a**) The main ones are models in which animals are induced to present a pathology similar to one that affects humans or other animals by administering drugs or other biologicals, inflicting injuries, or subjecting them to stress or other environmental conditions. In contrast, models based on spontaneous changes (**b**) include animals where the normal course of their life predisposes them to develop a specific disease. (**c**) Genetically-modified test subjects are animals with knockin or knockout genes or proteins. In contrast to using healthy animals (**e**), negative models (**d**) employ individuals that are not susceptible to certain diseases but serve to evaluate susceptibility to a specific pathology. TBI: traumatic brain injury.

Animal models are essential for several biomedical research fields such as cancer biology and therapeutics, neuroscience, pharmacology and toxicology, neurobiology of diseases, endocrinology, public health, palliative medicine, also, in studies in human and animal biology and for the discovery and testing of new drugs, vaccines, and other biologicals (e.g., antibodies, hormones) whose validation requires preclinical studies in animals [6,20]. Currently, these models address current research priorities, considered as those imposing major global threats to human and animal health. These include diseases that have afflicted humankind or increased exponentially in recent years such as SARS-CoV-2, different types of cancer and their therapy, cardiovascular diseases, metabolic and neurodegenerative disorders, and experimental refinement of surgical techniques to treat these issues [21]. The models may involve complete animals or only particular cells, tissues, organs, genes, or other agents that reproduce pathological processes (Figure 1) [8,22]. Species include rats, mice, guinea pigs, dogs, rabbits, birds, ruminants (cows, sheep), horses, fish, frogs, monkeys, cats, reptiles, squid, crabs, bees, chimpanzees, hamsters, sea slugs, pigs, nematodes (roundworm), fruit flies, and protozoans, among others [7].

The importance of animals in medical science is reflected, for example, in the percentage of Nobel Prizes studies in Physiology or Medicine using animal models (90%) [5]. From 1901 to 2020, two-thirds of those awards (186 of 222 projects [7]) employed animal models to understand pathogenic mechanisms, metabolic diseases, diagnostic and therapeutic procedures, develop vaccines, or test the efficacy of novel drugs [22]. At least 144 species used in those animal-based studies were mammals, and 42% were rodents [7]. Dogs were the first animal model used in metabolic research on gastric secretions [23] and for discovering insulin [24]. To date, rodents are the predominant species in research (Table 1) [9]. However, non-mammal species are trending, and the number of animals depends on the country and its legal regulation regarding the use and reporting of animals in research. Moreover, in some countries, there is no official annual report on animal research (e.g., South America), and not every country counts the same animals (e.g., the United States does not consider rats, mice, fish, birds, amphibians, reptiles, and cephalopods, they are not covered by the Animal Welfare Act). Although it might differ, Table 1 and Table 2 show an overview of the use of animals according to species in some countries and a summary of the reported statistics worldwide.

Several Nobel Prizes have been awarded for animal research, and the increasing number of animal models in different countries demonstrates these studies’ importance for scientific advancement [7]. However, just as necessary, their use also entails ethical challenges that require surveillance through laws, norms, guides, and strict bioethical committees to monitor the use and care of laboratory animals based on the principles of the 3Rs [33]. In this regard, for 50 years, the National Center for the Replacement, Reduction, and Refinement of Animals in Research (NC3Rs) has promoted Russel and Burch’s initiative of the 3Rs to reduce, replace, and refine procedures to improve the conditions of animals used in experimental protocols [34].

These norms differ from one nation to the next. However, one guide recognized internationally is ARRIVE (Animal Research: Reporting of in vivo Experiments), developed in 2010 to improve the in vivo experiments description to increase the reproducibility of results, refine the stages of study design, and clearly report the methods so they can be repeated and tested [35]. A second guide is PREPARE (Planning Research and Experimental Procedures on Animals), which seeks to determine and guarantee quality control in animal studies [36]. Today, for any experimental protocol requiring animals, proposals such as the Animal Study Registry (ASR) help researchers thoroughly plan their study design, methods, and statistical analyses to ensure transparency and reproducibility in their results [37]. Additionally, it is essential to mention that Ethic Committees must approve current experimental protocols within each institute to promote an appropriate use and care for animals in research.

Animal models certainly provide valuable information on the nature of diseases [38]. However, it is important to remember that inter-species limitations exist in anatomy, metabolism, physiology, and genetics [39], so a single preclinical model cannot represent all aspects of pathogenesis due to differences in resistance or susceptibility [38]. Currently, many animals used in biomedical studies undergo some genetic modification, such as transgenesis or the utilization of knockout or knockin genes, to visualize specific changes that would take years to develop under normal conditions [40]. Therefore, the selection of the animals depends on the specific research field; through their use, researchers develop scientific knowledge focused on human and veterinary medicine.

## 4. Animal Models and Their Application in Distinct Fields of Current Biomedical Science

### 4.1. Emerging Infectious Diseases

The SARS-CoV-2 virus is the etiologic agent of the coronavirus 2019 disease (COVID-19) [41]. This disease has claimed the lives of over 6.3 million people worldwide since 2019 [42,43]. The lack of knowledge of this virus and its rapid propagation at the onset of the pandemic made it essential to determine its physiopathology and identify therapeutic agents and vaccines that could mitigate its threatening consequences. These fundamental issues were solved using in vivo assays that replicated the virus in animals to untangle its pathogenesis, the immune response, and the adverse effects that might result from the vaccines and therapies proposed before testing in humans and their release to the public [41,44].

The choice of an animal model that would allow researchers to observe the histopathological, radiological, or immune changes that the virus caused required that the test animals be susceptible to lung tissue damage and capable of developing an inflammatory process [45]. Potential species included nonhuman primates, ferrets, rats, mice, Syrian hamsters, lagomorphs, minks, cats, camelids, and even zebrafish [46].

The transgenic mice can express the human angiotensin-converting enzyme II (hACE2), a functional receptor for the SARS-CoV-2 virus that mimics clinical signs observed in humans [47]. Sun et al.’s [48] research with 4.5–30-week-old transgenic mice successfully replicated the virus after intranasal and intragastric inoculation. It led to the discovery of viral loads in the lung, trachea, brain, and feces. Those authors also detected an immune and inflammatory response due to the presence of interleukins (IL). Adult mice showed more lesions in the alveolar epithelial cells, focal pulmonary hemorrhage, and more significant apoptosis of macrophages. Those findings concurred with human reports showing that COVID-19 affected older adults more severely, with the over-65 population representing 80% of all hospitalizations and a 23-fold greater risk of mortality. Reports emphasized clinical signs, such as respiratory distress and cytokine release syndromes [49]. Studies with Syrian hamsters found that while the virus is lung-tropic and infects the respiratory tract by binding to the ACE2 cell surface in the alveoli, causing pneumonia in 67% of the animals, the gastrointestinal signs reported in humans are due to viral replication and dissemination in enterocytes [50].

One animal model that shares multiple similarities with humans for the physiopathology of the SARS-CoV-2 virus is based on Rhesus macaques, African green monkeys (*Chlorocebus aethiops*), and crab-eating macaques (*Cynomolgus macaques*) [51]. The latter has been utilized to replicate the infection conditions in young (males and females of 3–9 years) and old-aged animals (23–29 years-old females). After intranasal and intratracheal viral inoculations, researchers found that nasal swabs (peak viral load of 10^6^ copies/µL) had higher viral loads than pharynx and rectal ones (a maximum of 10^4^ copies/µL). Additionally, viruses from nasal and pharynx samples were detected for longer periods in elderly monkeys [52]. This relation between age and disease mortality was also reported in Rhesus monkeys. Comparative studies of three nonhuman primates (three 3–5 years and two 15 years old macaques) infected intratracheally revealed that the viral replication detected by nasopharyngeal and anal swabs was persistently detected from 3 days post-infection (dpi) to 11 dpi in elderly animals. In older macaques, 104–107.5 copies/mL were also detected (while young individuals had approximately 104 copies/mL), often accompanied by the development of diffuse severe interstitial pneumonia [53].

The reinfection processes prevalent in human populations were replicated in studies with *C. aethiops*. Infection in six animals caused signs such as fever (50%), hypercapnia (66%), 2–7-fold increases in C-reactive protein concentrations (100%), and coagulopathy (100%) were recorded. That research proved that anal, oral, and nasal swabs could detect viral loads up to 15 dpi [44]. These findings are similar to those from other works with *M. mulata*, where viral RNA was found in swabs from the nose, pharynx, and anus, with amounts increasing up to 3 dpi (in an approximate range of 4–7 copies/mL) [53]. These nonhuman primate models undoubtedly contributed significantly to our understanding of the pathogenicity of COVID-19 and the physiological bases for implementing preventive and diagnostic measures and treatment.

Another important aspect of using animals is that they helped understand the transmission of the virus to other domestic species and showed that pets could acquire the SARS-CoV-2 virus through contact with an infected human. However, there is no evidence of active pet-to-human transmission [54]. Studies with dogs, pigs, chickens, and ducks showed they were not susceptible to COVID-19 infection due to low viral replication [55]. Identifying susceptible species made it possible to choose appropriate models for developing and testing vaccines [55]. Ferrets, Syrian hamsters, rabbits, transgenic mice [47], and cats were all found to be susceptible, the latter even vulnerable to airborne transmission with the development of clinical signs such as hair loss and pulmonary alterations similar to those seen in humans [56,57]. Apart from domestic cats, wild felines (tigers, lions, pumas, snow leopards) [58] have been reported to show infections by this virus. Kang et al. [59], who reported the first Delta variant (SARS-CoV-2 Delta) case in three domestic cats with COVID-19-positive owners in China, insist that transmission to pets is a topic of concern due to their possible role as silent intermediate hosts.

### 4.2. Endocrinology and Metabolic Pathologies

Obesity is a public health problem affecting over 600 million people worldwide [60]. Obesity and its associated metabolic syndromes have consequences such as knee osteoarthritis, a disease prevalent in approximately 60% of the overweight population [61], but this is also associated with cancer, cardiovascular disease, hypertension, coronary artery disease, stroke, sleep apnea, asthma, gallstones, steatohepatitis, and dyslipidemia. Over one-third of the world’s overweight or obese population is at risk of developing type 2 diabetes mellitus [23]. Using rodent models, researchers have determined that one element that promotes the development of type 2 diabetes mellitus is adipose tissue inflammation due to insulin resistance and excess fat mass [62]. The increase in the presentation of these comorbidities has led to the use of animal models to test new, improved strategies for reducing the incidence of this disease.

The role of the different types of adipose tissue in humans and animals is a crucial line of research that has developed with the use of rodents. For example, adipogenesis suppression and the browning of white adipose tissue (WAT) [63] have been suggested as strategies for preventing obesity [60]. The browning process creates a brown adipose-like tissue (BAT) that can participate in thermogenesis by transforming caloric intake into heat [64]. Since this is part of a central nervous system response to cold, certain medications and exercise can trigger browning as has been observed in obese and lean rats subjected to high-intensity training. In C57BL/6J mice, the transformation of beige adipocytes into WAT can be promoted with diets complemented with resveratrol for 16 weeks, as this induces a change in the intestinal microbiota in treated animals (*p* < 0.01) (increasing microorganisms of the genera *Bacteroides*, *Lachnospiraceae*, *Blautia*, *Lachnoclostridium*, and *Parabacteroides*, among others) that modulates lipid metabolism and has anti-inflammatory properties and anti-obesity effects [65].

The importance of physical activity in treating these conditions has been demonstrated in experiments with 48 Sprague-Dawley male rats, where aerobic exercise for 12 weeks combined with prebiotic fiber supplementation prevented knee joint damage, dyslipidemia, endotoxemia and normalized the effects of insulin resistance (*p* < 0.001) [61]. Studies with these supplements as part of a therapeutic protocol in Wistar rats, administered in presentations such as yogurt, have shown that supplementation with 5% of yogurt reduces levels of oxidative stress (significant decreases in NO levels, *p* < 0.05), and had fewer amounts of inflammatory cell infiltration and collagen deposits in the liver (*p* < 0.05) when compared to animals fed high-fat diets. According to these studies, this supplement could be a potential human therapeutic option [66].

Studies of the human genome have identified hundreds of genetic variants associated with obesity and opened the way to examining these genes in species such as *C. elegans*, a nematode capable of storing fat in the form of lipid droplets inside hypodermal and intestinal cells. *C. elegans* has 14 genes that promote diet-induced obesity and three that prevent it [67]. Those genes are now recognized as potential targets for anti-obesity treatment. Ke et al. [68] found that the knockdown of 23 fat-storing not only reduced excessive fat accumulation but also improved the health and lifespan of this species (*p* < 0.05). The inhibitory effect of flavonoids such as butein on lipogenesis in *C. elegans* succeeded in reducing triglyceride levels by up to 27% without altering food intake or energy expenditure, an effect due to the downregulation of proteins involved in lipid metabolism [69]. Likewise, the appetite suppressant effect of administering vegetable extracts from the *Lentinus strigosus* mushroom (300 and 1000 µg/mL) to *C. elegans* functioned as a natural means of preventing obesity [70]. Studies of this kind allow researchers to address obesity as a complex pathology affected by diverse factors: diet, physical activity, developmental stage, age, genes, and environmental interaction [67].

Another animal species considered a promising model for studying metabolic syndromes is the zebrafish (*D. rerio*). This species has genetic homology with humans, so through genetic mutation, chemical induction, and changes in diet, they can be used to study hyperglycemia, obesity, diabetes, and hypertriglyceridemia [71]. Pigs, meanwhile, share similarities with humans in terms of organ size, lifespan, anatomy, physiology, and metabolic profile [40]. A study of obesity in Iberian pigs showed the pathogenesis of chronic kidney disease caused by overweight and obesity. Although the administration of high-fat diets did not generate diabetes in those pigs by day 100, analyses revealed hypercholesterolemia (142 ± 27 mg/dl), hypertriglyceridemia (75 ± 43), insulin resistance, and glomerular hyperfiltration [72]. These effects also occur in humans [73] and have been studied in obese male mice and ovariectomized females [74].

The domestic dog has been postulated as a valuable model for studying chronic morbidities brought on by environmental conditions since they share morbidity and mortality factors with humans. In this field, Hoffman et al. [75] reported that comorbidities behind chronic conditions such as obesity, arthritis, hypothyroidism, and diabetes reported in humans were also present in 73,835 canines and that those dogs showed a positive association between age and the number of morbidities (*p* < 0.001). Other studies have revealed that obesity in dogs (137/198) is closely linked to the alimentary habits of their owners, finding that the 79.8% of dogs from overweight owners (114 persons) were obese (*p* < 0.001) [76]. Therefore, studies of these animals could provide information on disease interaction.

### 4.3. Cancer in Biomedicine

According to the World Health Organization [77] and the National Cancer Institute [77,78], the most common types of cancer in humans in 2020 were breast (2.26 million cases), lung (2.21 million), colorectal (1.93 million), prostate (1.41 million), skin (1.20 million), and stomach (1.09 million). These cancers cause 10 million deaths per year. Projections for 2022 estimate that around 1,918,030 new cancer cases will be diagnosed in the United States, with 350 cancer-induced deaths per day, making this disease a primary cause of mortality [79]. The pathogeny of these cancers and testing new treatment options is another field that extensively uses animal models. Over 95% of studies use rats and mice to inject cancer cell lines subcutaneously, study the primary cancer lesion, and follow its growth before excising tumors [80,81]. However, one disadvantage of this subcutaneous tumor model, is that injections in athymic nude mice may not accurately represent the interaction among tumor cells, local stroma, and the tumor’s microenvironment, depending on its precise location [82]. Contrarily, orthotopic murine models have been shown to replicate the tumor microenvironment –including metastasis– when inoculated in the original anatomical site of the tumor. In female BALB7c mice, inoculation of mammary cancer cell line 4T1 as a fat pad tumor model showed that 50% of the animals had metastasis to the ovaries, spleen, liver, and sternum. However, when compared to a heterotopic model, orthotopic tumors were smaller (1993.7 ± 197.15 mm^3^ vs. 1078.4 ± 300.26 mm^3^, *p* < 0.05) and had a significantly lower percentage of infiltrating cells (*p* < 0.05) [83]. Moreover, these orthotropic models, together with in vivo optical metabolic imaging, are proposed as an approach to studying how, for example, the fatty acid uptake by breast cancer cells increases accordingly to tumor aggressiveness and metastatic process (*p* < 0.05) [84] Attacking this complication in tumor development is the principal objective of anticancer therapies, since most deaths from prostate cancer, for example, are due to metastasis into bone structures [80].

Koosha et al. [85] used diosmetin, an anti-tumorigenic, in colon cancer xenografts in 24 male nude mice. Results showed that tumor volume in the group treated with 100 mg/kg of diosmetin was significantly smaller than in the untreated group (264 ± 238.3 vs. 1428 ± 459.6 mm^3^, *p* < 0.01). Promisingly, the drug did not produce toxicity even when administered at high doses. Studies of this kind show that laboratory animals allow researchers to test new drugs and better understand disease development but also aid in determining non-toxic doses that can be applied to humans or animals. Using these models as translational media for studying cancer has also revealed the importance of identifying the pain that animals may experience. Pain assessment is important in in human medicine and laboratory animal welfare. In this regard, recognizing degrees of cancer-induced bone pain has been studied by observing behavioral changes in rats and mice, where innate behaviors, such as burrowing, are reduced 9 days after inoculation when compared to control groups (*p* < 0.05) as a result of the nociception associated with the degree of severity of cancer due to reduced bone density [86].

The fact that the canine and human genomes share a high degree of similarity (75%) and that the risks of death due to neoplastic, congenital, and metabolic diseases are comparable means that the dog is an ideal translational model for studying human morbidity and mortality [75,87]. For example, the percentage of neoplasia is similar between dogs and humans (27.4 vs. 25.3%). However, because the types of cancer that affect each species correlate only marginally (Spearman rank *p* = 0.661) [75], dogs have been replaced in many preclinical studies by genetically-modified pigs [87].

Another novel anticancer strategy involves managing nerve-tumor interaction [88] since tumor-specific denervation can suppress neoplasia growth [89]. A study by Kamiya et al. [90] with female Balb/c-nu mice and the use of xenografts in Hras128 rats in a model of chemically-induced breast cancer showed that sympathetic stimulation of the nerves in tumors accelerated cancer growth but that parasympathetic stimulation reduced growth and downregulated the expression of programmed death. In contrast, in the case of late-stage colorectal cancer, parasympathetic denervation via vagotomy and atropine administration in 150 male Wistar rats reduced the incidence of tumors and their weight and volume after eight weeks, as well as cell proliferation, angiogenesis, and regulated expression of the nerve growth factor [89].

These neural anticancer therapies in humans and animals indicate that while sympathetic nerves show cancer-promoting effects in prostate and breast cancer, and melanoma cases, the parasympathetic/vagal nerves are believed to trigger both reactions. For example, vagal nerves can promote prostate, gastric, and colorectal cancers, but suppress breast and pancreatic cancers, due to β-adrenergic and muscarinic effects that modify the behavior of cancer cells, angiogenesis, tumor-associated macrophages, and antitumor immunity [88]. The axonogenesis process in species such as mice, linked to the development of metastasis in breast cancer, showed through immunofluorescence that nerve twigs tend to be sympathetic-like, with no expression of parasympathetic fibers [91].

In addition to the support of laboratory techniques such as immunofluorescence, non-invasive diagnostic methods are a priority in oncology. In immunocompetent genetically-engineered mouse models, Kirkpatrick et al. [92] utilized nanosensors with urine tests to detect protease activity in diverse types of cancer, including lung cancer, achieving 100% specificity and 81% sensitivity. In this way, monitoring with nanosensors and clinical assays in animals has demonstrated that this technique can be an option for conducting accurate, radiation-free diagnostic tests.

Nanoparticles and their application, together with in vivo imaging, can help to test novel luminescent particles and assess their tissue penetration to improve cancer therapy [93]. In vivo imaging enables us to understand tumor growth-related processes such as oxidative mitochondrial metabolism in mouse models with cell lung cancer [94]. Likewise, in a mouse model of brain tumor –glioblastoma– under general anesthesia, modified in vivo optical imaging (Surface enhanced spatially offset Raman scattering) covers the inability of conventional techniques that rely on subcutaneous inoculation of cancerous cells because they cannot read deep tissues [95]. These techniques are the basis for imaging-guided phototherapies that are a current research field to find agents capable of inducing tumor cell apoptosis, such as photodynamic y and photothermal therapy [96].

### 4.4. Pharmacology and Therapeutics

Parallel to the advances in our knowledge of the physiopathology of diverse conditions, developing and testing new therapeutic options is another field destined for animal models. Algology is a science in constant actualization to provide new and efficient drugs to prevent the consequences of pain by reducing the number and severity of secondary effects in both human and veterinary patients [97,98]. Adequate models are needed to evaluate analgesic efficacy accurately. In the case of treatments for open wounds, Parra et al. [99] applied carprofen (5 mg/kg) and buprenorphine (0.1 mg/kg) to the left hind paw of Sprague Dawley rats of both sexes using a punch biopsy to assess analgesia in an open wound model. Using four behavioral tests associated with aspects of nociception, mechanical and thermal stimulation, guarding behavior, and the weight-bearing test, they found that carprofen promoted recovery of the thermal response to basal levels after just 2 h. The same rat species were utilized to test the renal and gastrointestinal safety of non-steroidal anti-inflammatory drugs (NSAIDs) such as ibuprofen by administering single and multiple oral doses to pediatric patients. Furthermore, the necropsies performed on pigs of different ages (8-week-old and 6-to-7-months-old) in the study by Millecam et al. [100] revealed no severe lesions in the stomach after multiple doses of ibuprofen at 5 mg/kg. However, significant histological score differences (*p* < 0.025) were observed in the duodenum (1.38 vs. 4) and jejunum (3.63 vs. 1.25) between the experimental and control group. Additionally, an increased clearance time for the drug after multiple doses was found, an effect similar to reports in human pediatric patients.

Due to the adverse effects that NSAIDs can generate, especially for treating chronic afflictions such as arthritis and cancer, opioids are another therapeutic option [101]. However, since the long-term use of these drugs is also associated with complications, research has begun to new concepts and explore directions. The opioid-free anesthesia technique was introduced to prevent tolerance and hyperalgesia and reduce the use of these drugs in the postoperative period. This method uses agents such as alpha-2-agonists, ketamine, and local analgesics with distinct action mechanisms in multimodal analgesia [102,103,104]. Other new opioid-based pharmacological options are transdermal patches impregnated with morphine-like compounds. In 6–12-week-old C57BL/6JJmsSlc mice, patches synthesized with two new opioids (new-opioids 1 and 2, N1 at 3 mg/kg; N2 at 10 mg/kg) showed the same analgesic efficacy as morphine at 3 mg/kg. The effect remained constant, even under repeated administration (in contrast to fentanyl), and the cutaneous trans-permeability rate was greater, at 1.71 ± 0.35 and 3.94 ± 1.36 µg/cm/h [105]. The administration of opioid nanoparticles has also been suggested to prevent opioid tolerance and reduce the severity of adverse effects. Leucine-enkephalin hydrochloride-based nanoparticles with a size of 100–200 nm have been tested in male Sprague Dawley rats by applying them intranasally, reaching the brain directly. After dosing, high concentrations were found in the olfactory bulb and cerebrum between the first 60 min (approximately 80 ng/g and 160 ng/g, respectively), while plasma concentrations were not detected at any evaluation time (*p* < 0.0001). This prevents the side effects of drug transit through peripheral pathways [106].

Techniques based on local anesthesia temporarily relieve pain by inhibiting nerve impulse transmission. However, when used to complement multimodal analgesia protocols, they can be associated with neurotoxicity in both human and veterinary patients [107]. Administration via polymer-based encapsulation is a new strategy designed to prevent toxicity and permit the prolonged release of the active ingredient to give a long-term analgesic effect for up to seven days [107]. A ketamine-polymer-based drug was applied transdermally to Wistar rats to determine its analgesic effects [98]. Results of the tail-flick test and readings from an analgesiometer led them to determine a significant analgesic effect (*p* < 0.01) maintained for 24 h with a peak effect at 8 h and a response time on the test 5.72 s vs. a basal time of 2.44 s. The compound did not produce irritation when tested on rabbit skin. It prevented the secondary effects of intravenous, nasal, or oral administration, so it is a potential option for treating neuropathic pain [108].

### 4.5. Experimental Surgical Tecniques

In addition to developing novel drugs, advances in surgical technology and techniques have opened fields in microsurgery in human and animal medicine since the 1900s when Carrel and Guthrie performed the first transplants in dogs [109]. Later, in 1950–1960, Buncke and Schultz tested the first microsurgery techniques using models of digital amputations and reimplantation in Rhesus monkeys, performing vascular microsurgery to restore circulatory connections successfully [110]. Anastomosis of 1-mm blood vessels in the ears of adult rabbits by reimplantation was the first demonstration of microsurgery in reconstructive medicine [111].

Today, rodents are considered models for reimplanting extremities and restoring blood vessels because their vascularization is homologous to the human finger [112]. For example, developing heterotrophic osteomyocutaneus flap transplant protocols in Lewis rats furthered our understanding of the mechanisms and pathways involved in the immune response underlying tissue transplant rejection [113]. Likewise, in an experiment with five syngeneic mice and allografts—using a donor-supplied aorta and inferior vena cava—end-to-end anastomosis of those structures showed a 74% success rate as a technique for hind limb transplants [114]. In another study, Tee et al. [115] performed grafts of engineered cardiac muscle flaps in the epicardium of 8 rats. The flaps were transplanted by microsurgery to resolve one of the first limitations: failed vascular anastomosis. Those researchers performed successful end-to-end anastomosis of the carotid artery and jugular vein by placing the flap on the epicardium, achieving a survival rate of 75% during 4 weeks post-surgery, with viable cardiomyocytes and vascular connections between the flap and the epicardium by week 10 [115]. These techniques, tested first in animals, were later used with human patients with coronary artery disease caused by diseases such as squamous cell carcinoma, with a 96% survival rate of the flap in individuals subjected to neck and head surgery [116].

Another advance in biomedicine achieved thanks to experimental work with animal species such as pigs are based on animal-to-human organ transplants. On 7 January 2022, Bartley Griffith’s team performed the first heart transplant from a genetically-modified pig to a 57-year-old human patient with terminal heart disease [117]. Although the patient’s condition who received that xenotransplant deteriorated two months after surgery, and he died, the procedure set an important precedent. It showed the need to continue research on genetically-engineered animal organs and immunosuppressor drugs since the immune response and organ rejection are still the leading causes of transplant failure, especially when the organs come from other animal species [118].

Due to the physiological similarity between nonhuman primates and humans, procedures for organ transplants are often tested in those species. Over seven years, Lee et al. [119] performed 22 xenotransplants using hearts from transgenic pigs eliminating alpha-galactosidase transferase knockout or expression of the regulatory proteins CD46, CD39, or CD73 in Cynomolgus monkeys (*Macaca fascicularis*). Results showed that survival of the grafts was significantly higher in hearts with double or triple genetic manipulation (11.63 ± 11.29 days vs. 30.83 ± 20.34 days, *p* = 0.03). This is similar to the report by Cui et al. [120] on triple knockout cells from pigs (that do not express any of the three carbohydrate xenoantigens). The complement-dependent cytotoxicity response and the amount of anti-pig IgG/IgM immunoglobulins (Ig) were evaluated in serum from 72 specific pathogen-free (SPF) baboons and in human serum. Results for humans and old-world monkeys showed similar antibody binding, but the cytotoxicity measured in IgM and IgG was lower in the humans (*p* < 0.05 vs. *p* < 0.01).

Observations on the immunosuppressor response to compounds such as anti-thymocyte globulin (20 mg/kg) and rituximab (20 mg/kg) demonstrate that, in addition to the use of transgenic animals, a strict immunosuppressor regimen is a critical element in allotransplants [119]. In this regard, drugs injected in nanoparticles such as mycophenolate mofetil allow low-water soluble compounds to be combined with other compounds and administered as solid lipid nanoparticles to improve their absorption and release by as much as 68% in acid media [121].

In this field, sustained release options such as nanoparticle-anchoring hydrogel scaffolds of the immunosuppressant tacrolimus allowed the localized release of the drug with tissue regeneration in nude female mice or those of the BALB/c line that were given the drug in the hind limb. Those combinations allowed the sustained release of 77% of the drug, without toxicity, within 28 days at <100 ng/mL [122]. Thus, refining these drugs in the future will make it possible to reduce the cases of organ rejection due to the immune response. This finding is significant because their benefits are not accompanied by systemic toxicity, complications, or dose reduction without pharmacological efficacy [123].

### 4.6. Neurosciences

The field of neuroscience includes surgical and therapeutic procedures involving the central nervous system and conducts studies focused on specific diseases or pathologies of that system. With the discovery of neurological sequelae in COVID-19-infected patients, animal models have allowed researchers to observe the effects that the SARS-CoV-2 virus generates in sporadic cases, including epileptic seizures and encephalitis with a mortality rate of approximately 5.3% [124].

Estimates suggest that approximately 42 million people worldwide suffer brain injuries annually and that 80% of cases are classified as traumatic brain injury (TBI). Animal models based on rodent species are being used to improve our understanding of the physiopathology of TBI [125], though authors such as Vink [126] caution that neuroanatomical differences in the mouse’s lissencephalic brain can generate biomechanical responses distinct from those in humans. Moreover, the replication of trauma may be greater in rodents since traumatisms in these animals tend to generate focal instead of diffuse lesions [127]. Grovola et al. [128] used male Yucatán miniature pigs to analyze neurological dysfunction in animals with mild traumatism 1-year postevent. They found a persistent neuroimmune response in animals with morphological changes to the microglia, with increased branches and junctions per cell (*p* = 0.026 and *p* = 0.045, respectively). In other research, models of medullar lesions are widely utilized with species such as rats, which are particularly important because between 236 and 1009 per million humans annually suffer a spinal cord injury [129]. Although this species is the one most often employed to replicate medullar damage, Filipp et al. [129] affirm that between-species differences (quadrupeds, bipeds) must be considered when evaluating the neuroplasticity of the spinal neurons.

Epilepsy is one of the most common neurological conditions, affecting over 50 million people worldwide [130] and 0.6–0.75% of the domestic canine population [131]. Recent studies of the physiopathology of this disorder and the testing of anti-seizure drugs have used fruit flies (*D. melanogaster*) because they manifest seizure-like behavior and share 70% of their genes with humans [15]. The use of the endocannabinoid anandamide (at 2, 20, and 200 µg/mL) in *Drosophilas* prevented induced seizures (*p* < 0.0001). This led to the discovery that the action mechanism of their metabolites is not linked to the cannabinoid receptors but, instead, to transient potential receptors (TRP). This makes the fruit fly a suitable medium for studying this type of drug [132].

Despite its nature and supposed organic simplicity, *Drosophila* has been used to understand the neurobiological bases of processes still considered mysteries by biology, such as sleep, plasticity, and memory [133]. After studying 12,000 exemplars of *D. melanogaster*, Toda et al. [134] reported the existence of the “nemuri” gene, a peptide with antimicrobial properties that favors sleep and helps these flies survive the infection. This suggests that its function could be linked to the immune competence of the sleep process in animals and humans. The association of sleep with long-term memory, known as post-learning sleep, was studied by Lei et al. [135], who found a neural circuit that excites the mushroom body neurons and a connection to the fan-shaped ventral neurons that promotes post-learning sleep during courtship. This finding underlined the association between the longer learning experience and the reinforcement of long-term memory, mechanisms sometimes found in mammals.

Neuroscience techniques applied to species such as nonhuman primates and transgenic models of those species have recently been proposed as useful for studying human evolution and the cerebral functioning of people with autism disorders and neurodegenerative diseases such as Alzheimer’s [136]. In humans, Alzheimer’s disease is considered the most common neurodegenerative disease accounting for around 80% of cases of dementia worldwide [137]. It is widely recognized that mitochondrial dysfunction is an event that precedes the onset of Alzheimer’s, and this has been studied in two lines of mice (APPswe/PSEN1 ∆E9 and C57BL/6J). There, the alteration of mitochondrial homeostasis and increased mitochondrial calcium levels caused damage and neuronal death (*p* < 0.0001) due to deposits of amyloid plaques. Recognition of this physiopathology helped scientists establish the goal of preventing this process as a novel therapeutic approach [138].

Another neurogenerative disease, Parkinson’s, has been studied primarily with murine models [139]. Recently, however, researchers recognized that the zebrafish shares more neuroanatomical traits with humans and that mutations of the PARK7 gene in adult fish were associated with the development of Parkinson’s in humans [140,141]. Exposure of zebrafish larvae to neurotoxins that act directly on the dopaminergic neurons constitutes a method to mimic the phenotype of Parkinson’s disease. Specifically, the MPP+ neurotoxin affected the locomotor function (total distance and velocity) of fish, reducing its performance by 80% and 85%, respectively (*p* < 0.001). Furthermore, no systemic effects were observed, presenting a condition similar to Parkinson’s [142].

Palliative treatments to control movement disorders such as dystonia, Huntington’s, and Parkinson’s disease have also been tested in zebrafish [143]. Treatment of Parkinsonian embryos with substances such as rosmarinic acid (RA) prevents the loss of dopaminergic neurons due to neurotoxicity. This acid has been proposed as a neuroprotector and antioxidant that reduces locomotor deficits measured, for example, by increasing the swimming distance in zebrafish treated with RA at concentrations of 10 or 100 µm (approximately 130 to 150 cm, *p* < 0.01) [144]. Similarly, it has been suggested that herbal medicines based on Tongtian oral liquid have neuroprotective and antioxidant properties. The administration of Tongtian to zebrafish prevented neurotoxicity and the degeneration of dopaminergic neurons (*p* < 0.01 when compared to non-treated fish) while reducing larval behavioral impairment measured as improvements in the total distance (peak distance around 180 cm) and velocity (peak values around 3.5 cm/s) (*p* < 0.001) [145].

Aquatic models are also utilized to study other neurodevelopmental problems, such as autism spectrum disorder in zebrafish and Medaka fish (*Oryzas celebensis*) [146]. Chen et al. [147] found that prenatal exposure to valproic acid (at 5 and 50 µM) in AB lines of zebrafish produced embryos and larvae with signs similar to those seen in autistic humans, including hyperactivity, manifested in a greater frequency of tail-bending, greater distances traveled after touching of the dorsal tail (*p* < 0.001, *p* < 0.05), increased swimming speed under both light and dark conditions, and deficient social interaction, anxiety, and macrocephaly, all as consequences of neuronal cerebral cell proliferation. In a separate study, when applied to 28 neonate rat pups, this acid generated oxidative stress in the cerebellar hemispheres and reduced the count and nuclear size of the Purkinje cells [148]. These findings appeared, as well, in the brains of children with this condition. In the case of rats, administering grape seed extract served as a neuroprotector thanks to its antioxidant effect.

Referring to neurodegenerative disorders, a key strategy is to improve symptomatology through physiotherapy and rehabilitation protocols, another line of research that has increased in importance due to the prevalence of neurological conditions that can affect the quality of life of both humans and animals.

### 4.7. Physiotherapy and Rehabilitation

Because the number of neurodegenerative and traumatic diseases in humans and animals has been increasing in recent years, one of the main options for these cases is developing and implementing physiotherapy techniques. For example, stimulation of the lateral cerebellar nucleus with low-intensity ultrasound is a non-invasive therapy for reducing the consequences of cerebrovascular accidents in mice after induced ischemic stroke. In those test animals, functional asymmetry of the brain was restored, and pathological electrical cerebral delta activity was reduced, leading to improved performance on the beam-walking test [149].

In cases of osteoarthritis, for example, transcutaneous electrical nerve stimulation techniques (TENS) in physiotherapy protocols utilized in male Sprague Dawley rats with induced pain showed that when applied to the knee joint for 20 min a day for two weeks, TENS reduced the expression of c-fos (*p* < 0.05) (a biomarker of pain) on the day following the intervention (7302.80 ± 152.40% vs. 5074.50 ± 199.50%) in all the test animals that, in addition to TENS, did exercise on a treadmill (7333.40 ± 156.70% vs. 2790.00 ± 111.88%) [150]. In canine patients, functional neurorehabilitation after Hansen type I intervertebral disc surgery has been tested using a technique with bases similar to TENS called transcutaneous electrical spinal cord stimulation (TESCS). Combined with pharmacological treatment (4-AP) for 90 days, this approach restored ambulation in 88% of 16 animals thanks to the so-called multimodal neurorehabilitation protocol in a study by Martins et al. [151].

In human medicine, TENS has been used with patients with knee osteoarthritis. It improved performance on the stair-climbing test by 0.41 s [152] and reduced pain in individuals with head and neck cancer who had received radiation and developed oral mucositis with the pain. In those patients, 30 min of high-frequency TENS functioned as a non-pharmacological intervention that reduced pain levels at rest by approximately 3.0 from visit #1 to visit #3, as measured by the McGill Pain Questionnaire. However, this approach did not show results for controlling functional pain [153]. Pain reduction allowed the patients to exercise the limb and prevent the loss of mass, muscular strength, and joint instability with some cartilage recovery.

Electroacupuncture is a similar technique used to control chronic inflammatory pain. The action mechanisms of this technique have been studied in murine models after administering the complete Freund’s adjuvant to the hind paw. In those animals, electroacupuncture produced analgesia by attenuating neuronal signaling in the dorsal ganglia of the spinal cord, the anterior cingulate cortex, and neurons of the somatosensorial cortex. This suggests that the analgesia generated affects cortical pain pathways and means that the somatosensorial and anterior cingulate cortices may be potential therapeutic targets for developing new options for pain management [154], one of the principal objectives of rehabilitative medicine in humans and animals.

## 5. New Models and Strategies Applied in Animal Research

The use of poorly developed or unconventional species is expanding to other areas of biomedicine. For example, the zebrafish is used to study anomalies in limbs and craniofacial regions [155]. In those fish, Bergen et al. [156] found 604 genes associated with processes of the formation, mineralization, and regeneration of scales, which demonstrated that those structures are reminiscent of bone. Mutations of these genes in humans generate bone mineralization disease. This suggests that scales could be a model for studying the pathogenesis of skeletal diseases, calcification, and matrix formation [156]. In another fish species –Medaka, the Japanese rice fish (*Oryzias latipes*)– researchers found that the electrocardiogram pattern was more similar to that of humans than those of rats and mice. This led authors such as Yonekura et al. [157] to use it as a model for testing cardiovascular therapies and the response of action potentials to verapamil, which causes bradycardia, an effect also seen in humans [158].

In addition to the use of mammals such as domesticated dogs as models for research on urinary pathologies due to their anatomical and physiological similarity to humans [159], the diverse species that have been incorporated into biomedical science include protozoans, platyhelminths, planarians, cnidarians, bivalve mollusks, gastropods, cephalopods, annelids such as the tardigrades, and arthropods such as hexapods, crustaceans, arachnids, and various insects in studies in broad fields of investigation [160]. In dermo-cosmetology, extraction of hyaluronic acid from mollusks such as *Mytilus galloprovincialis* and *Crassostrea gigas* to treat wounds in Wistar rats accelerated the processes of wound repair and re-epithelization, allowing lesions to heal completely within 15 days of treatment, in contrast to the results attained with commercial healing creams [161]. Another application of a cephalopod (*Octopus vulguris*) is in reconstructive medicine due to its capacity to regenerate nerves and adjacent tissues such as muscle and blood vessels. Despite these technical advances s in medical research, additional studies are required to determine markers, antibodies, and imaging techniques designed to take advantage of those species [162].

Non-animal alternatives such as cell cultures, 3D tissue cultures or organs-on-chips, mathematical models, stem cells, bioprinting, in silico tests, and advanced computer simulations have been increasing in recent years [163]. In leading research countries and regions such as the United States, United Kingdom, China, Germany, Japan, Canada, and Australia, among others [164], there has been a particular interest in replacing animal models with another methods. This is promoted by ethical pressures, the 3Rs initiative, and official instances such as the National Institute of Health [165]. An example of this is the new US law sponsored by the Food and Drug Administration (FDA), which states that drugs no longer require animal testing before human clinical trials [166]. Another example could be Canada and the statistics regarding the number of rats and fish used as animal models from 2019 to 2020. In 2019, rats and fish went from 3.9% and 19.9% to 2.6% and 11.7%, respectively [26,167].

When mentioning tissue engineering, the so-called “organoids”—transplantable tissues created by engineering—have raised expectations for replacing animals, resolving specific bioethical issues by making the study of pathologies and drug testing more specialized [168]. Protocols for head and neck squamous carcinoma have been published, using patient-derived organoids to study therapeutic agents and their drug sensitivity [169]. However, as materials that depend on in vitro handling and do not come from organisms that provide blood flow or the biochemical conditions of a live individual, their development and clinical application require further advances, not only in medicine but also in applicable biotechnologies [168]. Current trials aim to establish the vascularization of organoids, such as in human brains [170] or kidney organoids., In vitro culturing under millifluidic chips and endothelial cells is an alternative to creating vascular networks that need future studies but can be an option to research nephropathies [171]. Complex vascular networks made with mesodermal progenitor cells by Wörsdörfer et al. [172] replicated the ultrastructure of a blood vessel in tumor organoids with endothelial cell junctions, luminal caveolae, microvesicles, and antiangiogenic responsiveness to stimuli. Moreover, 3D bioprinting of organoids derived from stem cells (e.g., ectoderm, mesoderm, and endoderm) is another alternative to replicate developmental diseases in the brain, skin, kidney, heart, intestine, lung, and liver [173]. Those biotechnological advances include approaches in which animal models are accompanied by artificial intelligence [174].

The support that robotics and artificial intelligence provide to the advance of science has improved the technologies involved in techniques of robot-assisted, minimally-invasive surgery [175]. Recently, machine learning techniques have been used with animal models to help diagnose or identify specific behavioral or physiological changes in species. In this regard, models of Parkinson’s disease in zebrafish have used video recording to teach the machine to differentiate between a movement disorder and a parkinsonian fish, a technique that may apply to cases of motor diseases in humans [140]. Deep learning algorithms, a type of machine learning, are another approach to the future of biomedical science, particularly in diagnosing a wide range of diseases. Based on CT images, it has been tested in hepatocellular carcinomas [176] and COVID-19 diagnosis, showing 85.2% accuracy a specificity and sensitivity of 88 and 87%, respectively [177]. A similar accuracy percentage (91%) was also obtained when testing deep learning to identify genetic syndromes according to facial features [178]. In veterinary medicine, automatizing facial recognition to assess pain is a current approach applied in cats, with an accuracy above 72% [179]. These applications suggest that new diagnostic tools might not require animal models. Nonetheless, implementing these technologies depends on their ability to simulate the physiology of a live organism, especially humans, to improve the replicability of results [180].

The replicability of animal models in preclinical protocols depends on their internal and external validity for transposing results to humans. However, the complexity of some human conditions and the physiological differences among species have led authors such as Pound and Ritskes-Hoitinga [181] to recommend focusing on techniques and technologies prioritizing human research. However, it is important to remember that experimentation with human subjects involves many serious ethical and legal controversies such as those surrounding experimentation with nonhuman primates [182]. One ethical way to deal with this topic consists in establishing and following norms and guidelines such as the 3R principles that promote the rational and humane use of laboratory species [33].

In summary, important advances in human and veterinary medicine have been mainly achieved thanks to animal species that allow us to improve our understanding of the etiology, pathology, physiology, and toxicology of diverse conditions that affect both humans and nonhuman animals [5]. However, using these species requires evaluating ethical considerations, existing limitations, the options available, earlier studies, and, above all, focusing on the welfare of laboratory species to fully recognize their enormous contributions to science.

## 6. Conclusions

Animal models—including a broad diversity of species of vertebrates and invertebrates—are a key element for experimental research aimed at replicating human and animal pathologies. Over the past five years, significant advances regarding worldwide priority diseases such as COVID-19, breast cancer, diabetes, obesity, and Parkinson, among others, were made in species such as nonhuman primates, rodents, lagomorphs, dogs, pigs, and even invertebrates such as zebrafish and nematodes. Moreover, before human clinical trials, novel therapeutic drugs, diagnostic techniques, and surgical procedures such as flaps or organ transplants have also been refined in animals.

These examples show the importance of using animals in biomedical research to study emerging or poorly understood human and animal diseases, and development of novel therapeutic options, including nanoparticles and in vivo techniques. Although animals will remain an essential element of science in the near future, due to their remarkable contributions, the ethical aspect of animal experimentation is significant.

The ethical pressure and the application of initiatives to reduce and replace the number of animals used in experimental protocols is leading to new strategies such as genetic engineering, artificial intelligence, organs-on-chips, mathematical models, bioprinting of organs, and advanced machine learning technologies. This multimodal approach is considered the best option for addressing the ethical dilemmas raised by using laboratory animals while emphasizing their valuable contributions to human and animal medicine.

## Figures and Tables

**Table 1:** Overview of the number of animals used in research, according to the species.

Country	Year	Specie	Percentage (%)	Reference
European Union	2019	Rodents	61.9	[25]
		Fish	24.6	
		Birds	6.2	
		Amphibians	0.5	
		Cephalopods	0.2	
		Dogs	0.1	
		Non-human primates	0.07	
		Other mammals	6.5	
Canada	2020	Birds	50.0	[26]
		Rodents	24.5	
		Fish	11.7	
		Cattle	11.3	
		Amphibians	1.1	
		Pigs	0.4	
		Dogs	0.2	
		Non-human primates	0.1	
		Reptiles	0.1	
		Other animals	0.5	
United Kingdom ^1^	2021	Mice	68.2	[27]
		Fish	12.9	
		Rats	6.5	
		Birds	8	
		Dogs	0.14	
		Non-human primates	0.09	
		Cats	0.01	
		Other animals	3.3	
United States ^2^	2019	Guinea pigs	23	[28]
		Rabbits	18	
		Hamsters	12	
		Non-human primates	9	
		Dogs	7	
		Pigs	6	
		Cats	2	
		Sheep	2	
		Other species	21	
South Korea	2017	Rodents	91.8	[29]
		Fish	3.3	
		Birds	2.3	
		Rabbits	1	
		Non-human primates	0.08	
		Amphibians	0.07	
		Other species	1.21	
		Total	3,085,259	

^1^ Excluding Northern Ireland; ^2^ Rats, mice, fish, birds, amphibians, reptiles, and cephalopods are not included.

**Table 2:** Approximate of the number of animals used in research worldwide between 2019–2020.

Country	Number of Animals	References
United States	20,000,000–24,000,000	[30,31,32]
China	16,000,000	
Japan	11,000,000	
European Union	9,400,000	
Australia	6,700,000	
Canada	5,067,778	
South Korea	4,141,433	
United Kingdom	3,300,000	
Norway	2,282,710	
Germany	2,151,805	
France	1,865,403	
Spain	761,012	
Mexico	685,315	
Switzerland	556,107	
Belgium	437,275	
New Zealand	240,000	

## Data Availability

Not applicable.
